# Spray Dried Formulations for Inhalation—Meaningful Characterisation of Powder Properties

**DOI:** 10.3390/pharmaceutics12010014

**Published:** 2019-12-21

**Authors:** Angelika Jüptner, Regina Scherließ

**Affiliations:** Department of Pharmaceutics and Biopharmaceutics, Kiel University, 24118 Kiel, Germany; ajueptner@pharmazie.uni-kiel.de

**Keywords:** pulmonary application, nasal application, filling process, vacuum drum, dosator, FT4 powder rheometer, flowability tests

## Abstract

Spray drying as a particle engineering technique is of increasing interest in the field of inhalation and is already being utilised e.g., for the PulmoSphere^TM^ products. As spray dried particles tend to agglomerate and are mechanically instable, low dose filling processes can be difficult. This study correlates powder flowability tests of spray dried formulations with filling processes with drum and dosator systems. Four pulmonary and four nasal powders with different characteristics in terms of shape, composition, and surface polarity were prepared and characterised for powder flowability according to Ph. Eur. and by powder rheometry. All formulations were filled with a manual drum TT and a dosator system. The classical flowability tests according to the Ph. Eur. showed a bad flow behaviour for hydrophilic pulmonary powders (x_50_ ~ 3 µm), whereas hydrophobic pulmonary particles and nasal particles (x_50_ ~ 25 µm) showed a better flowing behaviour. Powder rheometry supports this finding but can better differentiate flow behaviours.

## 1. Introduction

In drug delivery to the respiratory tract, the fundamental issue is to deposit the drug particles in the targeted tissues. For pulmonary application of dry powders, a very small particle size of below five micrometres in aerodynamic diameter is necessary [1]. For nasal application, however, a particle size > 10 µm is regarded as suitable in order to avoid a major share of particles being inhaled to the lungs [2]. Spray drying as a bottom up technology offers the possibility to develop formulations in the needed size range. Being a particle engineering technique, specific needs can be addressed and the particles can be designed accordingly and the formulation of sensitive APIs like proteins or peptides is possible as well [3]. Inhalable insulin, for example, was formulated as a dry powder by spray drying and distributed as Exubera^®^ [4,5]. It was a stabilised amorphous formulation and was advantageous to the aqueous solution in terms of storage stability, applicable mass, and low microbial growth [4]. Likewise, an enzyme formulation of superoxide dismutase, an antioxidant enzyme, which can be used in treatment of cancer, arthritis, and respiratory distress syndrome could be developed with spray drying [6]. For this purpose, the superoxide dismutase was induced into a liposome carrier and spray dried with disaccharide stabilisers, which resulted in an unaffected enzyme activity [6]. Furthermore, the possibility to create formulations for local high-dose and systemic delivery are promising aspects. For pulmonary high-dose delivery, e.g., needed for antibiotic therapy, spray drying may also be a versatile formulation technology. In the course of that, the PulmoSphere^TM^ technique is already being utilized, and products are available on the market [7,8]. Spray dried powders further allow for embed nanoparticles, creating nano-in-microparticle composites. This strategy can be used to administer drug nanocrystals of poorly water soluble drugs or polymeric nanoparticles, e.g., being loaded with antigen for mucosal vaccination, to the nose and the lung, respectively [9].

Unlike in standard formulations for inhalation, mostly comprising a large carrier which dominates the bulk powder properties such as density and mechanical stability, spray dried products consist of small particles only. Thus, the bulk material properties are governed by the spray dried product properties (small size, low density). Due to the overall small particle size of spray dried formulations for respiratory administration, the handling and processing of these powders are afflicted with difficulties. The large surface area leads to uncontrolled agglomeration and may hamper the filling process [10]. Further, spray dried materials are oftentimes less dense and of low mechanical stability, thus, the filling process itself can have a negative impact on the success of inhalation, as the powder gets compacted and may not be re-dispersed very well [11]. To avoid issues accounting for high cohesion, surface-active excipients such as L-leucine can be added before spray drying [12]. Alternatively, the particle morphology can be designed to reduce the contact area of the particles [3,13]. This approach is oftentimes realised with hollow spheres, sometimes corrugated, where low mechanical stability can be an issue during the filling process as the particles might be destroyed by mechanical stress. To gain insight in important powder properties critical to the filling process, previous studies evaluated coarse powders or interactive blends [14,15]. It was found that the bulk density, basic flowability energy, and wall friction angle had an influence on the fill weight of inhalation carriers. Fines as a more cohesive group of powders were also evaluated and found to be challenging to fill with the dosator system. This study represents an approach to characterise spray dried formulations of particle sizes needed for inhalation by means of classical flowability tests and powder rheology. Furthermore, the impact of filling-induced powder changes on aerodynamic behaviour is exemplarily for some formulations.

## 2. Materials and Methods

### 2.1. Preparation of Model Formulations

Pulmonary particles of an intended particle size < 5 µm were prepared by spray drying a 2% (*w*/*w*%) solution of mannitol (Roquette, Lestrem, France), erioglaucine disodium (0.5% calculated on the solid mass; Sigma-Aldrich, Saint Louis, MO, USA) and different excipients to adjust the morphology (Table 1). Erioglaucine disodium, also known as brilliant blue (BB), was used as a model “drug” substance in this study. Hydrophilic spherical particles (P/S/phil) were obtained by spray drying the solution using a Büchi Mini Spray Dryer B-290 (Büchi Labortechnik AG, Flawil, Switzerland) equipped with a high-performance cyclone and a two-fluid nozzle of 1.5 mm inner diameter. The inlet temperature was set to 130 °C, and the outlet temperature was adjusted with the liquid feed pump rate to 64 °C. The Mini Spray Dryer was operated at an aspirator flow rate of 100% (35 m^3^/h) and an atomising air volume of 470 m^3^/h. To induce a wrinkled morphology in hydrophilic particles (P/W/phil), 0.02% (1% calculated on the solid mass) hydroxypropylmethylcellulose (HPMC 4000, Shin-Etsu, Wiesbaden, Germany) was added to the solution. For hydrophobic spherical particles (P/S/phob) leucine (Sigma-Aldrich) was added in a moderate concentration of 0.2% to the solution. Hydrophobic wrinkled particles (P/W/phob) were prepared by increasing the leucine concentration to 30% calculated on the solid mass. The spray drying conditions were the same for all pulmonary formulations.

Larger nasal particles (targeted particle size was 20 µm–30 µm) were likewise prepared in four different varieties. Hydrophilic spherical particles also consisted of mannitol and BB but were spray dried from a 10% (*w*/*w*%) solution. The BB content was 0.1% (N/S/phil) calculated on the solid mass. Wrinkled hydrophilic particles (N/W/phil) had 1% HPMC 4000 added to them. Hydrophobic spherical particles (N/S/phob) had 10% leucine calculated on the solid mass added and the wrinkled (N/W/phob) 18% leucine. The solutions were spray dried with the Büchi Mini Spray Dryer B-290 and a 60 kHz ultrasonic nozzle set to 1.6 Watt to create larger droplets and a lower aspirator rate of 50% to increase the drying time. An inlet temperature of 130 °C was set and the outlet temperature was adjusted with the pump rate to 60 °C. All powders were stored in a dry desiccator to avoid uptake of humidity after preparation and to allow after drying.

### 2.2. Bulk Characterisation

#### 2.2.1. Morphology

Morphology was evaluated by scanning electron microscopy (SEM; Phenom XL, Phenom-world; Eindhoven, the Netherlands). The samples were prepared by fixing the particles onto a carbon sticker and sputter-coated with a 20 nm gold layer. A backscattering detector and a working voltage of 10 kV were used.

#### 2.2.2. Particle Size Distribution (PSD)

The PSD was assessed with a HELOS laser diffractometer (Sympatec GmbH, Clausthal-Zellerfeld, Germany) with the RODOS dispersing system. Pulmonary particles were dispersed at 3 bar and nasal particles at 0.2 bar to ensure particle integrity. Data analysis was performed by the Windox 5.8.0.0 software from Sympatec (Clausthal-Zellerfeld, Germany) utilising the Fraunhofer theory. The reported data is the mean value of three-fold determination. The span value was calculated with the following Equation (1):

Span = (*x*_90_ − *x*_10_)/*x*_50_ (1)

#### 2.2.3. Contact Angle (CA)

The contact angle (CA) was determined on a pressed specimen (achieved with a hydraulic press PW 10 from Paul-Otto Weber GmbH, Remshalden, Germany) of the produced material with a mixture of water/glycerol (70:30), as pure water could not be measured due to high capillarity of the hydrophilic formulations. The liquid was dropped on the surface of the pressed specimen and the mean angle of contact between the surface and the droplet (measured with a goniometer Type G1 from Krüss GmbH, Germany) is reported as the mean of six measurements.

#### 2.2.4. Specific Surface Area (SSA)

Utilising the Brunauer-Emmett-Teller-equation the specific surface area was determined with a Gemini 2360 (Micromeritics, Norcross, GA, USA) and nitrogen as testing gas. Mean values were calculated of three measurements of the multiple point BET and reported with the standard deviation.

#### 2.2.5. Bulk and Tapped Density

The bulk density (BD) was assessed by determining three times the volume of 25 g (Sartorius BP 3100 S, Göttingen, Germany) formulation in a 100 mL measuring cylinder with an accuracy of 1 mL. The cylinder was tapped 10, 500, 750, and 1250 times on an Erweka SVM (Erweka, Langen, Germany) to obtain the tapped density (TD). If the volume did not change more than 1 mL in between the 750 and 1250 taps, the volume of 1250 taps was reported as the tapped volume (according to the Ph.Eur. 9.4 (2.9.34)). If the change was more than 1 mL, the sample was tapped repeatedly in 1250 steps until the specification was met. The ratio of the mass and tapped volume was calculated as the tapped density. To determine compressibility the Hausner ratio (HR) and compressibility index (CI) were calculated and evaluated according to the Ph. Eur.

#### 2.2.6. Flow Through an Orifice (FtO) and Angle of Repose (AoR)

The flow through an orifice was determined by placing a metal hopper containing 20 g of formulation on a conical orifice with a diameter of 25 mm in a granulate flow tester (GTB Granulate Flow Tester, Erweka). A vessel to collect the outflowing powder was set on a balance and the time was measured for the powder to pass through the orifice. For very poor flowing powders, it was possible to stir the powder. The data was reported as the mean of three measurements in s/100 g.

The angle of repose was tested with the granulate flow tester (Erweka) equipped with a 25 mm orifice in case of very cohesive powders (P/S/phil and P/W/phil) and 15 mm orifice for better flowing powders. In a hopper above the nozzle, the formulation was stored and flowed onto a base with a fixed diameter. The determination of the repose angle was done with the assistance of a laser and repeated three times, so the mean value could be calculated.

### 2.3. FT4 Measurements

#### 2.3.1. Powder Stability Measurements

Powder stability was assessed using an FT4 Powder Rheometer (Freeman Technology, Worcestershire, UK). Seven identical test cycles with a blade running at a speed of 100 mm/s downward through the sample were conducted and the energy was measured, which was needed to stir the powder. The ratio of the energy of test 7 and test 1 was deduced as the stability index (SI). An index of 0.9–1.1 indicates a robust material, which is not affected by stirring [16]. The measured energy of test 7 was taken as the basic flow energy (BFE), which is needed to overcome the resistance of the powder to flow [17]. To test the sensitivity of the powder, a test sequence with different blade speeds followed the stability test. The speed was reduced to 70, 40 and 10 mm/s, the Flow Rate Index (FRI) was calculated as the ratio from the energy of the last test and the energy needed for the 100 mm/s in the beginning. An FRI ~ 1 indicates that a powder is insensitive to change in flow rate. Most powders exhibit an average flow rate sensitivity of 1.5 < FRI < 3.0 and very cohesive powders a high sensitivity with an FRI > 3.0 [18]. During the upward movement of the blade, the specific energy (SE) was recorded as a measure of powder flow in a low stress environment. At an SE < 5, the powder is of low cohesion, between 5 and 10 of moderate cohesion, and an SE > 10 results for highly cohesive powders [17,18,19].

#### 2.3.2. Powder Compressibility

Compressibility measurements were carried out with a vented piston. A vertical stress in steps of 0.5–15 kPa was applied and the change in powder bed height due to the powder compaction was measured. The compressibility was calculated as a percentage change in volume [20] and classified according to the Carr’s index (CI, Ph. Eur. 9.4). Cohesive powders have a high compressibility due to agglomeration and large amounts of air entrapped in the bulk.

#### 2.3.3. Shear Cell Measurements

Shear cell tests were conducted with the 1 mL shear cell to achieve an understanding of the flow behaviour of a consolidated powder. The measurement starts with a conditioning step to ensure the same packaging in every sample. Afterward, a pre-compression with a vented piston is applied to obtain reproducible results with well-defined failure points [21]. This is followed by diving of the shear head into the powder bed generating a vertical stress. If the required stress is established, the shear head starts to rotate and induces the shear stress. The maximum shear stress, just before the powder bed fails, is recorded as the yield point. Plotting the yield points against their corresponding normal stress allows determination of the major principle stress and the unconfined yield strength. The ratio of the major principle stress and the unconfined yield strength is reported as the flow function coefficient (ffc) [21,22]. An ffc < 4 indicates a cohesive powder, an ffc of 4–10 an easy flowing powder and > 10 a free flowing powder according to Jenike’s classification [22,23].

### 2.4. Filling Trials

#### 2.4.1. Drum TT

**Figure 1:**
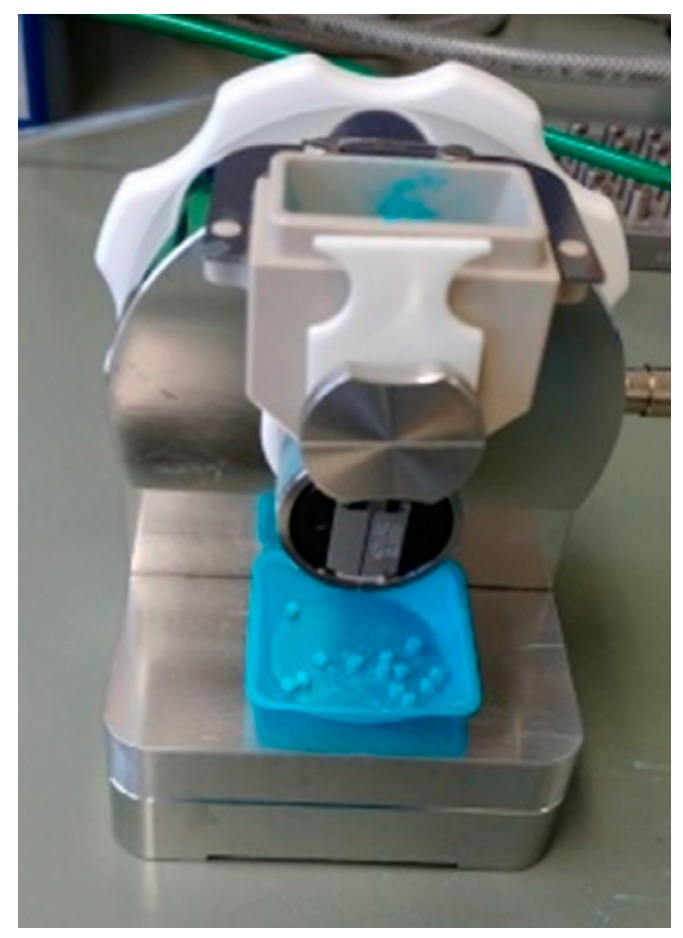
Picture of the used Drum TT equipment (Harro Höfliger).

The Drum TT (Harro Höfliger, Allmersbach im Tal, Germany) is a manually operated lab table unit for dosing trials, which can be equipped with a drum with different dosing bores (Figure 1). In this study, dosing bores with a volume of 15 mm^3^ for pulmonary formulations and 30 mm^3^ for nasal formulations were used, which were filled by the applied vacuum. As a maximal vacuum of −0.6 bars could be selected and −0.4 bars were set as moderate filling condition. To support the filling process, a stirrer implemented in the hopper above the dosing bore was turned manually three times. The ejected plugs were weighed (Sartorius analytical balance), and the mean value of 20 doses and the relative standard deviation (RSD) were calculated. A filling trial was considered uniform at an RSD < 10%.

#### 2.4.2. Dosator

**Figure 2:**
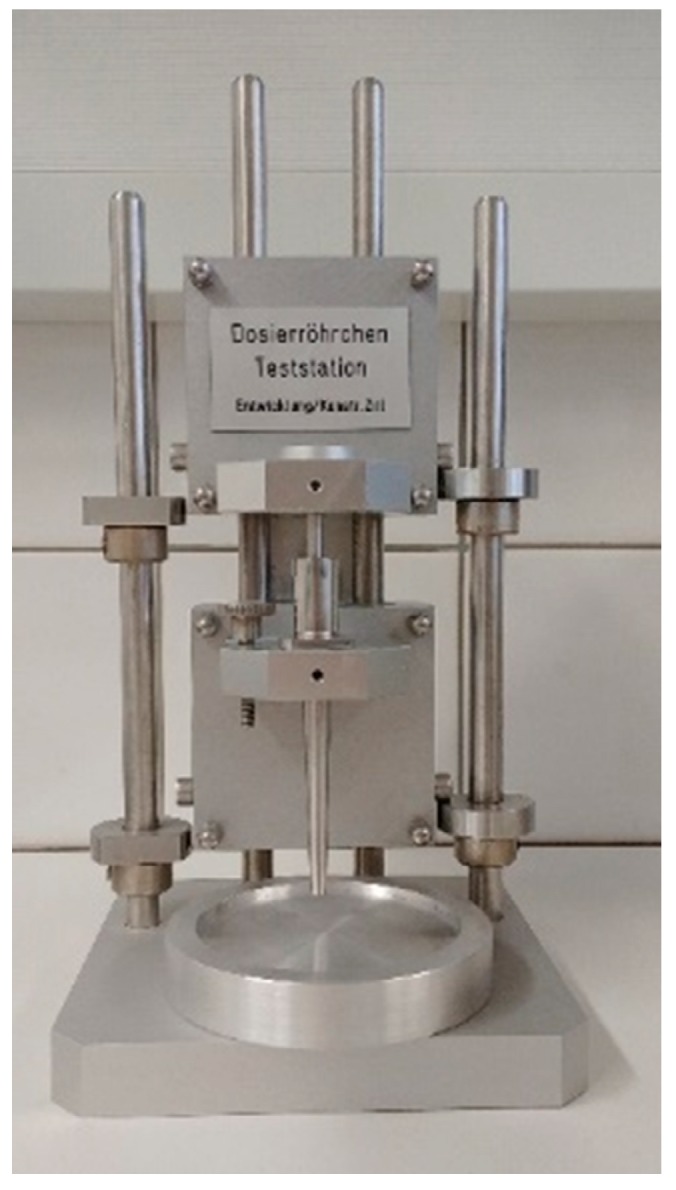
Picture of the used dosator system (Harro Höfliger).

To simulate the filling process with another filling system, a dosator table test station was utilised (Figure 2). A solitary pin suitable to fill capsules size 3 was implemented. To obtain a homogeneous powder bed with a smooth surface excessive powder was removed from the reservoir in every direction before a dose was taken with the pin, resulting in a 3 mm powder bed height. The powder had been sieved beforehand to destroy large agglomerates hampering the powder bed preparation. The pin separated one dose at a time from the bulk and compacted it to 1 mm height. In this manner, 20 doses were taken, and the mean plug weight and RSD were evaluated.

#### 2.4.3. Aerodynamic Performance of Dosed Powder Plugs

For aerodynamic characterisation, the emitted dose and fine particle fraction for chosen formulations were determined. The test of emitted dose (ED) was derived from the Ph. Eur. 9.4. A flow rate assuring a 4 kPa pressure drop over the device was set, that was 55.0 L/min for the Twister^®^ device (Aptar Pharma, Le Vaudreuil, France). Five individual doses were emitted from the Twister^®^ device without the device being cleaned in between the shots. The emitted dose was collected in the dose uniformity sampling apparatus, and the amount of brilliant blue, being present in all particles, was quantified via UV/Vis-spectroscopy at a wavelength of 630 nm.

The fine particle fraction (FPF) was assessed according to the Ph. Eur. 9.0 (2.9.18) with a next generation impactor (NGI) at a flow rate as described previously. To retain the powder in the collection cups the cup surface was coated with a mixture of Brij 35, ethanol, and glycerol (15/51/34%, *w*/*w*). A filter was set after the multi orifice collector (MOC). Quantification was conducted as stated above and data evaluation was performed with the Copley Inhaler Testing Data Analysis Software (Copley Scientific, Nottingham, UK). The percentage of particles of the emitted dose below five micrometres is determined as fine particle fraction (FPF).

## 3. Results and Discussion

As the pulmonary and nasal formulations differ in particle size, even though all are micronised powders, an improved flowability was anticipated for the nasal formulations due to their larger particle size. This should be reflected in the classical flowability test according to the Ph. Eur. and the FT4 measurements. Further, it was expected, that indented particles would show a worse flow and filling behaviour, because the indentations could interlock. A hydrophobic surface could improve flow and filling characteristics due to weaker particle-particle interactions. For powder filling, especially for low doses as common in respiratory delivery, powder flow and powder compressibility are important parameters determining the success of powder filling. Thus, spray dried powders for respiratory delivery will be especially challenging to fill. Further, the filling process is anticipated to have an influence on the ED and FPF, as the built-up plugs may not be well dispersed, and large agglomerates could remain in the device.

### 3.1. Characterisation of Bulk Properties

Spray drying resulted in particles in their respective targeted size range (Table 2). Pulmonary formulations had a particle size < 5 µm with a comparable size distribution, whereas the nasal formulations were >10 µm [2] due to a higher concentration of solution used and the use of an ultrasonic nozzle.

**Figure 3:**
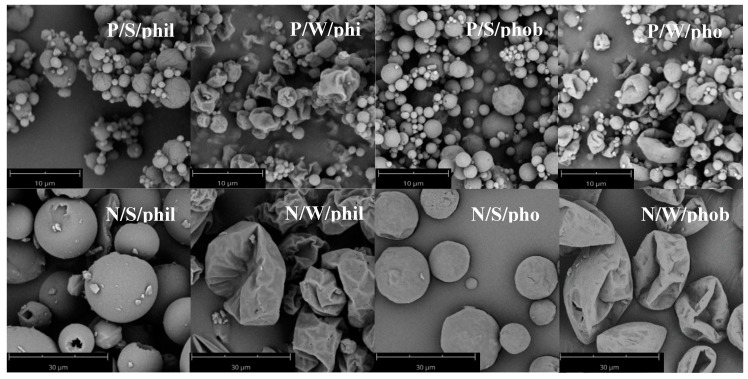
Scanning electron microscopy (SEM) images of pulmonary formulations in the top row. SEM images of the nasal formulations in the bottom row. Magnification of 5000× used for pulmonary formulations and magnification of 500× used for nasal formulations.

Leucine is known to form a shell in the early drying stage which leads to a hydrophobic surface and can alter the shape of the particles [12]. Measurements of the contact angle (Table 2) are in agreement with this. All powders can be moistened as the measured CA is below 90°. The addition of HPMC as hydrophilic excipient did not have an influence on the CA. Leucine as a hydrophobic amino acid with an aliphatic sidechain, in contrast, is poorly soluble in water. With this hydrophobic element being added to the powder, the CA increased to 85°. As the CA increased further the more leucine was added, this seems to be concentration-dependent. A change in morphology from spherical to indented (Figure 3) was induced as well by leucine. Particles consisting of mannitol and brilliant blue exhibited a spherical morphology. Adding 10% leucine did not alter the spherical appearance but the surface was slightly more uneven than for the particles not containing leucine. Increasing the leucine amount, the particles collapsed and became corrugated. HPMC had the same effect as leucine with respect to indentations, but its appearance was different. The area around large indentations was more structured with smaller wrinkles than those of the indented particles with leucine. Consequently, the surface area increased for the wrinkled particles compared to the spherical ones. Likewise, a decreased bulk density was to be expected. This could be observed for the hydrophobic particles, whereas the hydrophilic particles showed a higher tendency to agglomerate; thus, the effect of the morphology on the density was cancelled out.

According to the classification of the Ph. Eur. regarding HR and CI, the pulmonary powders exhibit poor to very, very poor flow characteristics (Table 2). The larger particle size and thus, less entrapped air in the bulk decreased CI and HR for nasal formulations. Their flow behaviour was fair to very poor. Wrinkled hydrophobic formulations can be compressed the best according to their HR and CI, as the particles are not aligned properly at first, but can be arranged through tapping better than their spherical counterparts can.

Whereas the bulk and tapped density is a quite static measure, which also highly depends on the measurement settings, AoR describes the result of an actually moving/flowing powder bed. However, the AoR turned out not to be a suitable characterisation method for the spray dried powders. Due to a bad flow behaviour, a larger orifice than the recommended orifice of 10 mm (in DIN ISO 4324) had to be used. Moreover, the powders had to be forced through the orifice. The fact that for pulmonary hydrophilic powders a large orifice of 25 mm had to be used and in the other cases a smaller orifice sufficed of 15 mm shows a trend to better flowability for hydrophobic formulations and powders with larger particle size.

The more dynamic test of flowing through an orifice showed similar problems and likewise a trend to a decreased flowability of small, wrinkled, hydrophilic powders (P/W/phil). The formulation had to be forced through the orifice the indentations could interlock. This test also allows for a rough estimation of powder flow for micronised spray dried powders.

In summary, the result of these flowability tests being proposed by the Ph. Eur. was the confirmation of unsuitability of these tests for spray dried formulations for pulmonary and nasal applications. They are able to show a trend for flow characteristics but cannot make precise distinctions. Therefore, instead of these tests, which are meant for freely flowable powders, studies with an FT4 powder rheometer were conducted. It conducts dynamic tests with standardised movement. With this the powder stability, sensitivity to variable flow rates, compressibility, basic flow energy, wall friction angle, and flow function coefficient were determined.

### 3.2. Results of FT4 Measurements

Prior to conducting the powder rheology measurements, a stability test was typically carried out to get an idea of whether the powders may change their flow characteristics during testing or processing. Cohesive powders can undergo agglomeration and change the flow behaviour during testing [16]. Therefore, the stability index was determined. The sequence of identical tests allows detecting changes in powders and the cause for them.

All tested powders exhibited a stability index (SI) above 1 indicating an instable powder bed (Table 3). The reason for this instability may be de-aeration and formation of larger agglomerates [16]. Due to the escaping air, the particle packing was denser, and consequently, more energy was needed to stir the powder as the particles were in closer contact with each other. The entrapped air seemed to escape better due to fewer agglomerates from the bulk in hydrophobic powders, causing an increase in the energy needed to stir the powder. The interlocking of the indentations in case of wrinkled particles further hampered the stirring resulting in higher SIs.

Additionally, compressibility can change flow behaviour leading to caking and an increased energy needed during stirring and hence, formation of an instable powder. Other factors like moisture uptake and incurrence of electrostatic charge can as well lead to enhanced agglomeration and raise the SI. For nasal formulations with a broad particle size distribution, segregation can occur and change the measured energy as smaller particles are packed more efficiently [16].

The BFE test measures the energy needed to establish a flow pattern producing a high stress flow mode in the powder in a downward rotation through the powder bed [17]. A relation of the flow pattern to a given process is not necessarily possible as the powder is confined in the vessel and is being pushed downward to the vessel floor. Therefore, compressibility is a factor, which may influence the result [24]. Further, the BFE is dependent on many variables. These factors are particle size, PSD, cohesiveness, density and moisture content, to mention some [17]. Therefore, nasal formulations with a larger particle size and mass will require more energy to be stirred than pulmonary formulations (Table 3). The overall needed energy is very low due to the low density resulting from hollow spheres, compared to e.g., a finely milled lactose of 20 µm with a BFE ~ 600 mJ [24]. Hydrophobic particles needed less energy to be stirred, due to less and less stable agglomerates compared to hydrophilic formulations; meanwhile, a wrinkled morphology can hamper the flow as observed for N/W/phil. The increased BFE of hydrophilic spherical pulmonary particles compared to the wrinkled particles was observed, because the agglomerates of P/S/phil required a higher stirring energy compared to the interlocked wrinkled particles.

The FRI was measured as the processing steps oftentimes include stirring motions at different speeds. The powder being subjected to this may be sensitive to different flow rates, so gaining an understanding in that aspect is important. All pulmonary powders except P/S/phil exhibited a high sensitivity to change in flow rate and required a higher energy to be stirred at lower stirring speed. This may be caused by less air in the bulk at low flow rates and the material being more consolidated locally by the blade [18]. This is an often-observed behaviour of cohesive materials. Nasal particles exhibited an intermediate sensitivity to change in flow rate.

**Figure 4:**
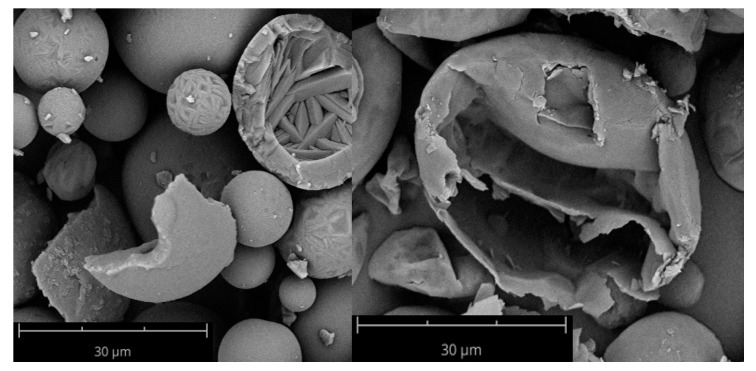
SEM images of N/S/phil (left) and N/W/phob (right). Magnification of 2500× was used. A difference in wall thickness can be seen.

Comparing the compressibility indices of the tapped density as per Ph. Eur. with the FT4 measurements, the same trend—larger particles are less compressible—was seen but the actual values differ for some samples, which can be explained by the differences in the measurement procedure. During the FT4 measurement, the powders are conditioned to ensure the same packing of the particles. Therefore, the FT4 measurement is more objective and reproducible, whereas the measurement with a cylinder as per Ph. Eur. is more dependent on the user and used cylinder. Under maximal pressure of the vented piston of the FT4 (a piston that allows the air to escape through a steel net), the influence of the morphology could be observed, as the compressibility of wrinkled particles was lower than for spherical particles due to their shape independent of the surface. The unusual good compressibility of N/W/phob could be explained by possible fragmentation of the particles and a resulting reduction of volume. Particles containing leucine can be thin walled due to the formation process with leucine precipitating fast at the surface and forming a coherent shell restricting the water vapour to the core [12]. As the pressure increases, the coherent shell is expanded resulting in thin-walled collapsed particles [12]. Consequently, the mechanical stability can be reduced. This can be observed in Figure 4.

The overall very good compressibility of spray dried powders has to be taken into account in processing steps such as storage in bins or hoppers as a solidification can take place.

A correlation between the SE and ffc was observed in agreement with literature [24]. The SE is measured during an upward rotating motion of the blade, with no consolidation of the powder. Thus, the shearing is depending on cohesion, particle size, shape and texture [19,24]. The ffc is a measure of flow of a previously consolidated powder, but for both tests, the sample was not further compressed during the test as it was in the BFE measurement. As the SE is a measure to evaluate the flow in a low stress environment, e.g., when the powder is being fed gravimetrically during die filling [19] compared to forced die filling, this can apply as well for ffc. The hydrophilic particles showed higher SE values, which indicated a moderate cohesion, and lower ffc values, indicating the same. Hydrophobic pulmonary powders and hydrophilic nasal powders exhibited similar SE values and low cohesion. In accordance, the ffc value was higher and classified them as easy flowing. The SE for large hydrophobic particles was even lower and the ffc indicated the powders are free flowing. However, the ffc measurements showed a more detailed differentiation in flow characteristics with respect to morphology differences than the SE, as the latter showed just the same tendency.

Powder rheological tests conducted with the FT4 proved to be more suitable for spray dried powders. They all showed similar trends in terms of cohesiveness and flow characteristics. The flowability of the powders was best described by the ffc.

### 3.3. Results of Filling Trials

Filling trials were conducted with a vacuum drum and a dosator system, which both fill volumetrically. The filling process was evaluated and tried to correlate to one of the flowability tests described previously with the aim to be able to predict the fillability of powders with a certain filling system based on a simple flowability test. During the filling trials, it turned out that not all formulations could be successfully filled with the filling equipment used.

#### 3.3.1. Drum Filling

The drum system offers the possibility of low dose filling and typically is a filling process with a low variability. A usual RSD in filling with the vacuum drum is <3% [25]. Filled doses depend on the size of the dosing bore and the used vacuum. In literature, interactive blends were filled with a vacuum of –0.4 bars representing mild filling conditions [26]. As flowability is especially impaired for hydrophilic pulmonary formulations, it was expected that these powders are either not fillable because of the occurrence of arching in the hopper of the drum, which is a common problem for cohesive powders [27], or will be filled with high variability.

Results showed, that hydrophilic pulmonary powders were filled with a higher variability (RSD ~ 6%) than usually seen for the drum system (Table 4).

All formulations could be easily filled with the drum except the hydrophilic pulmonary powders. Here uncontrolled agglomeration, adhesion to walls and arching occurred in the powder container. Consequently, the variability of these filling trials exhibited an RSD > 3%. The filling of these powders was repeated, and the success of filling and the variability was found to be dependent on the occurrence of these phenomena. As the filling trials were conducted under ambient environmental conditions, factors like humidity are still under investigation. Pulmonary hydrophobic and nasal powders were filled easily with RSD < 2% without arching or rathole formation. A wrinkled morphology seemed to interfere with filling trials, as RSD was higher for all wrinkled samples except hydrophilic pulmonary powders.

**Figure 5:**
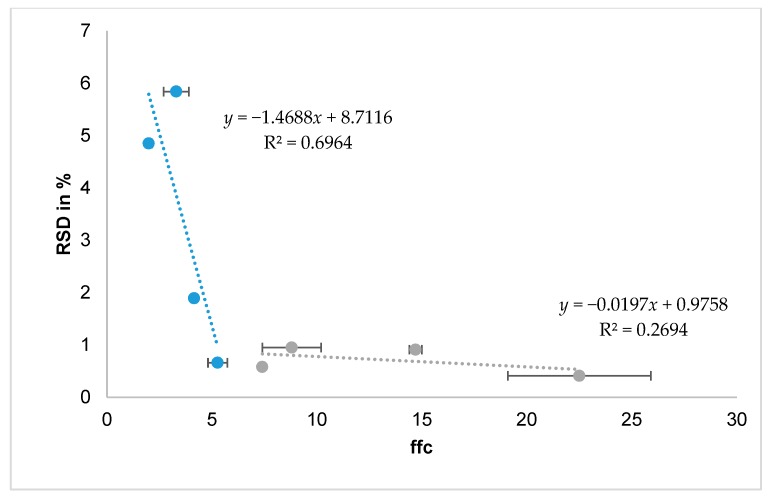
Correlation of RSD of filling trials with drum system and ffc as flowability measure. Blue dotted line represents the regression line for pulmonary and the grey dotted line the regression line for nasal formulations (*n* = 20).

The best characterisation methods to predict fillability with the drum system seem to be the ffc and SE. Powders with low cohesion and therefore good flowability are fillable with the drum system, whereas an ffc < 4 and SE ~ 5 indicate poor flow behaviour and are not reproducibly fillable with the drum system, as arching and uncontrolled agglomeration obstruct the filling process. A correlation of the RSD and the ffc could be found for the pulmonary formulations but not for the nasal formulations, as they all are fillable (Figure 5).

#### 3.3.2. Dosator filling

The dosator system is an established filling system for all kinds of powders. A dose is separated from the powder bed, compacted, and retained in the pin. Plug formation in the pin is needed to allow powder transfer to the capsule. The plug formation depends on the compaction and arch formation, therefore large particles with excellent flow behaviour and the inability to form the needed plugs cannot be filled with the dosator system [14].

In terms of spray dried powders, some challenges occurred. Filling of hydrophilic formulations was successful, whereas hydrophobic formulations displayed a more challenging filling behaviour. Pulmonary hydrophobic powders could be compacted but not retained in the pin and thus could not be filled. It has been described in the literature that cohesiveness is an important factor in dosator filling trials [14] and apparently the particle cohesion in those formulations was not intense enough to keep the powder plug stable. Nasal hydrophilic powders had a better powder flowability resulting in more dense powder beds. At the same time, they showed increased cohesiveness due to their hydrophilic surface compared to their hydrophobic counterparts, as seen from higher SE values, which allowed the formation of stable plugs. Larger spherical and hydrophobic particles (N/S/phob) showed excellent flowability in shear cell measurements and had a comparably high bulk density. Thus, the prepared powder bed in the reservoir was very uniform and dense allowing the dosator to separate and compact a large powder mass, which appears to be beneficial in this setting. For nasal wrinkled particles, the indentations could hamper a uniform powder bed preparation with density differences in the powder bed and therefore resulted in unsuccessful filling in terms of RSD. For larger powders, the formation of a uniform and dense powder bed in the reservoir seems to be key to successful filling. All wrinkled formulations showed a higher RSD that could result from an inconsistent powder bed, as the particles with indentations could not be aligned as well as spherical particles. Furthermore, filling trials with the dosator system showed an overall higher variability than filling trials with the drum system. A possible reason for this could be the general difficulties in uniform powder bed preparation. It has to be mentioned that sticking of powder as a thin layer to the outer wall of the pin was noted in some cases. The vibration during the critical process steps (powder ejection), however, was not enough to shake the powder off and influence the fill weight as it is a manually operated testing station. In a faster filling process, this might be a problem, which needs attention.

A correlation of the ffc and SE with filling success was found for pulmonary powders in the way that small powders with an ffc > 4 and SE < 5 resulted in an unsuccessful filling session. As nasal powders all exhibited good flow behaviour with an ffc > 4 and could be filled successfully with the dosator, the particle size seems to have a major influence. The fill weight showed no clear correlation to bulk or tapped densitym as the extent of compression during the powder bed preparation by removing excessive powder could not be taken into account. Other correlations, such as particle size and compressibility as proposed for different powders [14], were not observed. The slightly different powder bed preparation and filling process compared to literature [14,25] could have caused this. The main factor for the mass filled into the capsules in the present filling process is the uniformly packed powder reservoir and powder bed height, which was prepared manually. In literature, the powder bed is de-aerated ensuring a homogeneous powder packaging. Further, the volume of the dosing chamber is pre-set through the height of the inner piston in the pin. The pin dives into the powder bed causing a pre-compression of the powder. Therefore, a correlation can be found in the literature that smaller particle size and high compression increase the fill weight [14].

Overall, it appeared that the better flowable powders were easy to fill with the vacuum drum system, whereas the more cohesive powders exhibited some difficulties in filling the bore consistently. It has to be kept in mind that those filling trials were performed with table-top, single dose versions of the respective equipment, which was operated at low speed. Simultaneous multiple dosing at production speed may impose different challenges.

### 3.4. Aerodynamic Performance

Capsules filled manually with a spatula and capsules filled with the filling systems were assessed for their aerodynamic performance to evaluate the potential impact of powder change in dosing. To avoid an influence of differing fill weights between manually filled and machine-filled capsules, the fill weight of the manually filled capsules was adjusted to the machine-fill weights (i.e., 9 mg for P/S/phil and 12 mg for P/S/phob). The emitted dose from the inhaler and the fine particle fraction based on the emitted dose were investigated. It was anticipated that the filling processes could have an influence on the aerodynamic performance as the consolidation of the powder into a plug in the filling process could hamper the dispersion of the formed plug from the inhaler device. Consequently, the emitted dose and/or fine particle fraction could decrease.

As an example, capsules filled with hydrophobic pulmonary formulations were assessed in terms of emitted dose comparing manual and drum filling (as they were not fillable with the dosator system). Manually filled capsules of P/S/phob had an emitted dose for the Twister^®^ device of 66 ± 3% (mean value of five consecutive shots ± standard deviation). Capsules filled with the drum system resulted in emitted doses of 64 ± 6% for the Twister^®^ device. There is no statistically significant difference in the emitted doses, but a trend is seen to slightly lower emitted doses for both inhalers if the powder is filled with the drum.

The fine particle fraction of manually filled capsules of P/S/phob for the Twister^®^ device was determined to be 72 ± 2%. A statistically significant reduction to 67 ± 1% (*p* = 0.005) was observed for capsules filled with the drum system. This can be a result of a hampered dispersion from the device due to the compression of the powder to a plug.

The hydrophilic pulmonary formulation P/S/phil being filled with the dosator system showed no statistically significant difference in the emitted dose assessment compared to manually filled capsules. P/S/phil had an emitted dose for the manually filled capsules of 61 ± 5% and dosator filled capsules of 65 ± 21% (both from the Twister^®^ device). As the mass variability of the dosator-filled capsules was very high, this also impacted the emitted dose result.

The fine particle fraction for P/S/phil of manually filled capsules and the Twister^®^ device was found to be higher than for the dosator-filled capsules. A reduction from 39 ± 2% (manually filled capsules) to 35 ± 6% (dosator-filled) was observed. Furthermore, the variability increased with the dosator-filled capsules as the dispersion from the plugs was hampered. Visual inspection of the devices further revealed that dosator powder plugs were not dispersed completely, but larger fragments instead of fine deposited material was left in the device. This did not alter the overall deposition profile nor device retention in the current study but indicates that dispersing forces in the Twister were not intense enough to fully disperse the plugs.

Overall, it can be concluded that the filling process may have an influence on the aerodynamic performance, so careful investigation is needed.

## 4. Conclusion

In this work, spray dried powders for pulmonary and nasal application with their respective particle size were produced, characterized, and filled with the drum and dosator system. It could be shown that the classic flowability characterisation according to the Ph. Eur. is not suitable for the powders as results were not discriminative nor predictive for powder behaviour in filling. Powder rheometer measurements can discriminate better in terms of flow behaviour, especially the ffc and SE. These values can also be applied to predict the success of filling trials for the drum system. Other factors like BFE and compressibility showed no correlation to the filling trials but may be helpful parameters to gain deeper insight into powder characteristics. Filling processes can also have an impact on the aerodynamic performance, which should be taken into account.

## Figures and Tables

**Table 1:** Overview of composition of developed formulations for pulmonary and nasal application.

	P/S/phil	P/W/phil	P/S/phob	P/W/phob	N/S/phil	N/W/phil	N/S/phob	N/W/phob
Solid content in spray dried solution (*w*/*w*)	2%	2%	2%	2%	10%	10%	10%	10%
Concentration brilliant blue (*w*/*w*) in particles	0.5%	0.5%	0.5%	0.5%	0.1%	0.1%	0.1%	0.1%
Concentration mannitol (*w*/*w*) in particles	99.5%	98.5%	89.5%	69.5%	99.9%	98.9%	89.9%	81.9%
Concentration of added excipient (*w*/*w*) in particles	-	HPMC 4000 1%	Leucine 10%	Leucine 30%	-	HPMC 4000 1%	Leucine 10%	Leucine 18%

**Table 2:** Characterisation of pulmonary formulations (left) and nasal formulations (right). All values (except CA) are the mean of a threefold determination ± standard deviation. The CA values are the mean of a six-fold measurement ± standard deviation.

Parameter	P/S/phil	P/W/phil	P/S/phob	P/W/phob	N/S/phil	N/W/phil	N/S/phob	N/W/phob
PSD in µm	2.73 ± 0.02	2.92 ± 0.02	2.74 ± 0.03	2.66 ± 0.06	23 ± 0	26 ± 1	23 ± 0	27 ± 1
Span	1.86	1.86	1.91	2.08	1.01	1.93	2.44	1.69
CA in °	26 ± 4	33 ± 3	67 ± 9	85 ± 3	28 ± 4	25 ± 2	69 ± 4	81 ± 5
SSA in m^2^/g	1.88 ± 0.02	2.18 ± 0.04	1.55 ± 0.11	3.28 ± 0.07	0.38 ± 0.03	0.86 ± 0.04	0.96 ± 0.08	0.95 ± 0.04
BD in g/mL	0.23 ± 0.00	0.27 ± 0.00	0.36 ± 0.00	0.26 ± 0.00	0.36 ± 0.01	0.49 ± 0.01	0.47 ± 0.01	0.24 ± 0.00
TD in g/mL	0.33 ± 0.01	0.39 ± 0.00	0.56 ± 0.00	0.44 ± 0.02	0.48 ± 0.01	0.60 ± 0.00	0.66 ± 0.00	0.37 ± 0.01
HR	1.45 ± 0.02	1.43 ± 0.02	1.54 ± 0.01	1.70 ± 0.07	1.32 ± 0.03	1.23 ± 0.01	1.39 ± 0.03	1.56 ± 0.05
CI in %	31 ± 1	30 ± 1	35 ± 1	41 ± 2	24 ± 2	18 ± 0	28 ± 1	36 ± 2
Classification of flow according to Ph. Eur.	Poor	Poor	Very poor	Extremely poor	Slightly poor	Fair	Poor	Very poor
AoR in °	37 ± 3	46 ± 1	41 ± 2	40 ± 2	36 ± 1	31 ± 3	20 ± 3	34 ± 3
Fto in s/100 g	37 ± 5	148 ± 45	1 ± 1	2 ± 0	4 ± 3	1 ± 0	1 ± 0	2 ± 0

**Table 3:** Characterisation of pulmonary and nasal formulations with FT4 Powder Rheometer. All values are mean values of a threefold determination ± standard deviation.

Parameter	P/S/phil	P/W/phil	P/S/phob	P/W/phob	N/S/phil	N/W/phil	N/S/phob	N/W/phob
SI	1.30 ± 0.04	1.35 ± 0.14	2.71 ± 0.24	4.60 ± 0.58	1.31 ± 0.08	1.14 ± 0.11	1.28 ± 0.05	1.37 ± 0.22
BFE in mJ	38.7 ± 3.72	23.6 ± 1.85	14.93 ± 0.21	19.50 ± 2.35	31.6 ± 1.21	54.1 ± 0.65	22.89 ± 0.64	15.55 ± 0.77
FRI	2.46 ± 0.41	3.31 ± 0.71	4.87 ± 0.21	3.82 ± 0.19	2.37 ± 0.06	1.63 ± 0.01	2.21 ± 0.12	2.28 ± 0.14
SE in mJ	4.85 ± 0.67	5.11 ± 1.59	3.51 ± 0.57	3.56 ± 0.19	3.19 ± 0.19	3.23 ± 0.11	1.80 ± 0.03	2.10 ± 0.05
ffc	3.30 ± 0.6	1.99 ± 0.16	5.27 ± 0.46	4.15 ± 0.03	7.4 ± 0.1	8.8 ± 1.4	22.5 ± 3.4	14.7 ± 0.3
Compress (15 kPa) in %	48 ± 2	41 ± 1	47 ± 6	42 ± 2	22 ± 1	18 ± 0	16 ± 0	33 ± 1

**Table 4:** Fill weights with standard deviation and relative standard deviation (RSD) of *n* = 20 dosing procedures for the dosator and drum system. n.a. = not available, if the filling could not be conducted.

	P/S/phil	P/W/phil	P/S/phob	P/W/phob	N/S/phil	N/W/phil	N/S/phob	N/W/phob
Fill weight in mg **drum system**	6.10 ± 0.36	7.78 ± 0.38	11.92 ± 0.08	9.57 ± 0.18	17.37 ± 0.10	18.50 ± 0.17	19.37 ± 0.08	13.49 ± 0.12
RSD in %	5.84	4.85	0.66	1.89	0.58	0.95	0.41	0.91
Fill weight in mg **dosator system**	9.89 ± 0.5	9.81 ± 0.66	n.a.	n.a.	16.12 ± 0.67	24.36 ± 1.16	20.40 ± 0.81	7.27 ± 1.15
RSD in %	5.03	6.70	n.a.	n.a.	4.16	4.77	3.98	15.77
